# Knowledge about cervical cancer and HPV immunization dropout rate among Brazilian adolescent girls and their guardians

**DOI:** 10.1186/s12889-020-8410-9

**Published:** 2020-03-06

**Authors:** Ana Carolina da Silva Santos, Nayara Nascimento Toledo Silva, Cláudia Martins Carneiro, Wendel Coura-Vital, Angélica Alves Lima

**Affiliations:** 1 Programa de Pós-graduação em Ciências Farmacêuticas (CiPharma), Escola de Farmácia, Universidade Federal de Ouro Preto, Campus Universitário, Morro do Cruzeiro, Ouro Preto, Minas Gerais 35.400-000 Brazil; 2 Programa de Pós-graduação em Ciências Biológicas, Núcleo de Pesquisa em Ciências Biológicas (NUPEB), Universidade Federal de Ouro Preto, Ouro Preto, Brazil

**Keywords:** Human papillomavirus (HPV), Cervical cancer, HPV vaccine, Vaccine uptake, Knowledge, Adolescent girls

## Abstract

**Background:**

Infections with Human Papillomavirus (HPV) are the main cause of cervical cancer. Since 2014, the HPV vaccine was introduced in the Brazilian National Vaccination Calendar. The purpose of this study was to assess the knowledge of adolescent girls and their mothers/guardians about HPV and HPV vaccine, identify the factors associated with this knowledge, and evaluate immunization dropout rate.

**Methods:**

This was a cross-sectional study involving adolescent girls and their mothers/guardians. Participants underwent an interview that addressed sociodemographic data, sexual and gynecological history, and knowledge about HPV, HPV vaccine and cervical cancer. The third quartile of the total score was established as a cutoff for assessing knowledge. Adolescents who correctly answered more than four questions and mothers/guardians who obtained more than five correct responses were categorized into high knowledge. Poisson regression analysis was performed to identify variables associated with low knowledge. Vaccination records were used to assess immunization dropout rates. Any adolescent who did not complete the two-dose vaccination schedule was considered dropout.

**Results:**

A total of 666 adolescent girls and 623 mothers/guardians were interviewed. Low knowledge was observed in 76.7% of adolescents and 79.8% of mothers/guardians. Most were unaware of the causal relationship between HPV and cervical cancer, signs and symptoms of HPV infection, and had limited knowledge about the HPV vaccine. Factors associated with low knowledge of adolescents were aged 12 years [IRR 1.2 (95% CI 1. 1-1.3)] or less [IRR 1.3 (95% CI (1. 2-1.4)]; household income lower than US$750 [IRR 1.7 (95% CI 1. 1-2.6)] and household income between US$751 and US$1500 [IRR 1.6 (95% CI 1.0–2.6)]. Among mothers/guardians, low knowledge was related to having completed elementary school or less [IRR 1.5 (95% CI 1. 2-2.0)]; and household income lower than US$750 [IRR 1.2 (95% CI 1.0–1.4)]. Knowledge of adolescents and mothers/guardians was not associated with vaccine uptake. HPV immunization dropout rate was considered high (32.3%).

**Conclusion:**

Knowledge about HPV and cervical cancer as well as vaccine uptake was low. Results highlight the need for educational interventions about HPV and cervical cancer. These actions may contribute to improve adherence to HPV vaccination.

## Background

Cervical cancer is the fourth most prevalent type of tumor and the fourth cause of cancer death in women. In 2018, it was estimated 569,847 (3.2%) new cervical cancer cases and 311,365 (3.3%) deaths worldwide [1]. This cancer is more incident in developing countries, because of less access to prevention, screening, and treatment programs [1]. In Brazil, cervical cancer is the third most common cancer and the second cause of death by cancer among women. In 2018, it was estimated an incidence of 15.43 cases per 100,000 Brazilian women [2].

Persistent infection by high-risk Human Papillomavirus (HPV) is the main cause of cervical cancer [3]. More than 160 HPV types have been characterized and about 40 can infect anogenital tract and other mucosal epithelia [4, 5]. HPV6 and 11, classified as low-risk, account for most cases of genital warts. Conversely, high-risk HPV types, particularly HPV16 and 18 are associated with 70% of cervical cancer and precancerous cervical lesions [6].

The highest rates of HPV infection are observed in women less than 25 years of age [7]. A survey conducted in the United States with women aged 14 to 65 years showed that sexually active adolescents aged 14 to 19 years (35%) presented the highest prevalence of high-risk HPV infection [8]. In São Paulo, Brazil, the prevalence of high-risk HPV infection in women aged 15 to 65 years was 17.8%, with a higher rate among women under 25 years (27.1%) [9]. A study conducted in Rio Grande do Sul State, Brazil, including women with a mean age of 32.7 years demonstrated that patients aged 20 years or younger were at increased risk of HPV infection [10]. In Ouro Preto, Minas Gerais, a similar study showed a high prevalence of HPV infection in women younger than 30 years old (17.2%), who also had a risk of HPV infection approximately four times higher than women aged 50 years and older [11].

To reduce the cases of cervical cancer and HPV-related diseases, prophylactic HPV vaccines have been developed as a primary prevention strategy. The bivalent vaccine protects against HPV16 and 18, and the quadrivalent vaccine protects against HPV types 6, 11, 16 and 18 [12, 13]. Currently, the 9-valent HPV vaccine is available in the United States and includes an additional five HPV types namely HPV 31, 33, 45, 52 and 58 [13]. HPV vaccines are preferentially recommended for adolescents aged 9 to 14 years because this group had been less exposed to the virus through sexual intercourse and have shown the greatest immune response [12, 13].

In 2006, the United States, Australia, and Canada were among the first countries to implement HPV vaccination, and by the end of 2016, HPV vaccine was introduced into the national immunization programs in 74 countries [14]. Quadrivalent HPV vaccine was provided free in Brazilian routine vaccination schedule since 2014. In the first year, the target population was adolescents girls aged 11 to 13 years. Currently, HPV vaccine is available for girls aged 9 to 14, and boys aged 11 to 14 [15].

HPV vaccines are considered safe and effective for the prevention of HPV infection [12, 13]. However, a decrease in uptake has been observed since the incorporation of HPV vaccine in Brazil. In 2014, vaccine uptake for the first dose was 85%, but for the second dose, there was a reduction to 60%. In 2018, uptake to the initial dose remained high (79.2%), but there was also a drop in adherence to the second dose (48.7%) [15, 16]. Studies have shown some barriers regarding vaccination such as the belief that adolescents are too young to receive a vaccine against a sexually transmitted infection (STI), concerns about adverse effects of the vaccine, and lack of information about the HPV vaccine [17–19]. Knowledge about HPV and HPV vaccine varies in the general population and is an important factor in the acceptance of vaccination [17, 20]. Thus, some studies have been carried out to assess the knowledge of the population in several countries about HPV, HPV vaccine, and cervical cancer [21–23]. However, to the best of our knowledge, no study to date has evaluated the knowledge among under 15 years girls in Brazil, the main target population of HPV vaccination.

The present study aimed to assess knowledge of adolescent girls and their mothers/guardians about HPV, HPV vaccine and cervical cancer, and also identify the factors associated with this knowledge, and to evaluate the immunization dropout rate. The results may contribute to health promotion actions aimed to improve adherence to vaccination and other HPV prevention methods to reduce HPV incidence and other STI.

## Methods

### Ethical statement

This study was approved by the Human Research Ethics Committee of Universidade Federal de Ouro Preto (protocol number 858,572). All procedures in this study are in accordance with the resolution number 466/12 of the Brazilian National Health Council.

### Study design and participants

A cross-sectional study was conducted in Ouro Preto, state of Minas Gerais, Brazil, between 2014 and 2016.

Ouro Preto is a small town with a population of 70,281 inhabitants, and 36,004 (51.2%) are women. It is located about 100 km from Belo Horizonte, the capital of the state of Minas Gerais [24].

The study population consisted of adolescents who were between 11 and 13 years old in March 2014. March was used with reference, as this was the month that started the free HPV vaccination program in Brazil for adolescents of this age group. In addition to adolescent girls, their mothers/guardians were included in the study.

Sample size calculation was based on a population of 1750 adolescent girls between 11 and 13 years old. A list of name, age, and address of adolescent girls and their mothers/guardians was provided by Municipal Health Service. Prevalence of knowledge and perception about HPV and cervical cancer as “Good/Very Good” was estimated to be 13%. This prevalence was defined in a pilot study carried out with adolescent girls and mothers enrolled in two Basic Health Care Units of Ouro Preto.

Considering a confidence interval of 95% (95% CI) and an error of 2%, a sample size of approximately 670 adolescent girls was obtained.

### Data collection

With the support of health care teams, adolescent girls and their mothers/guardians were visited at their residences in urban and rural areas or were invited to attend the nearest Basic Health Care Units. The recruitment of some adolescent girls also took place in schools. The adolescent girls and mothers/guardians were informed about the purpose of study and invited to participate in the research. The adolescent girls and mothers/guardians who agreed to take part in the study all signed the assent and informed consent, respectively. All adolescent girls also needed an authorization of their mother/guardian obtained by form of written consent. Interviews were conducted by female undergraduate and graduate students who received prior training to perform this function. Adolescent girls and mothers/guardians were individually interviewed ensuring privacy. The mean time of each interview was 15 min.

Two specific interviews were developed, one for adolescent girls and another for mothers/guardians. The items in the interviews were elaborated based on previous surveys conducted in Brazil and other countries, considering the aim of the present study and local specificities [17, 25–28]. This instrument was piloted with adolescent girls and mothers attending two Basic Health Care Units. The wording of the interviews was modified based on the comments of the participants, aiming to improve understanding and ensure the validity of the responses. The two interviews contained similar items, being composed by multiple-choice and open questions that addressed: sociodemographic data, sexual and gynecological history, as well as knowledge about HPV, HPV vaccine, and cervical cancer.

In the knowledge assessment section, initially the participants were asked if they had heard of HPV vaccine and then of HPV infection. Adolescent girls and mothers/guardians who reported had never heard of these subjects did not answer the corresponding questions and were classified as low knowledge. Only those who answered affirmatively about awareness of HPV vaccine were asked to respond to the following open questions: How many doses of HPV vaccine should be given? What is the interval between HPV vaccine doses? Who should get HPV vaccine? Does HPV vaccine prevent other sexually transmitted infections?

Similarly, those who answered “yes” about awareness of HPV infection were invited to respond to specific questions on this topic, such as: What is the main transmission route of HPV? What should women do to prevent HPV infection? Does HPV infection have visible signs and symptoms? What are the signs and symptoms of HPV infection? Is HPV infection common? What kinds of problems does infection with HPV cause? What causes cervical cancer? Why should we prevent HPV infection?

Immunization records were used as data source to investigate the HPV vaccination uptake. These records were made available by the immunization sector of Ouro Preto’s Municipal Health Service.

### Data analysis

Interviews were coded and doubly typed in EpiData software, version 3.1. Subsequently, validation of double-entry, conference, and correction of divergences of typing was performed. Data analysis was carried out using Stata/SE software, version 14.

Answers to open questions were categorized according to their similarities and were later classified as correct or incorrect. Participants were given one point for each correct answer, and zero for incorrect answers or “do not know”. These points were added up and the knowledge was categorized as high or low. For categorization, the third quartile (Q3) of the total score was established as a cutoff. Finally, knowledge was classified as high according to the following criterion: adolescent girl - more than four correct answers in a total of 13 questions; mothers/guardians - more than five correct answers in a total of 10 questions. As the interview of adolescent girls and mothers/guardians had a different number of questions, it was necessary to transform the absolute number of correct answers in percentage to compare the grade of knowledge between the groups. This percentage was calculated according to the number of correct answers of each participant based on the total questions that assessed the knowledge of each group. The comparison of knowledge between the groups was performed using the Mann-Whitney test.

Poisson regression with robust variance estimation was performed to evaluate factors associated with knowledge of adolescent girls and the mothers/guardians using incidence rate ratio (IRR) with 95% CI. Initially, univariate analysis was carried out and variables with *p* value < 0.25 were selected. The univariate model was composed by all sociodemographic and behavioral variables, and vaccine uptake. The variables selected in this process were grouped in a model with successive discard (backward selection) of the non-significant variables, thereby considering the effects of these variables on knowledge. Thus, only variables with *p* value < 0.05 remained in the final multivariate model.

Vaccine uptake was based on the receipt of two doses of HPV vaccine and was assessed by the immunization dropout rate. Thus, it was considered dropout the adolescent girl who received only one dose of HPV vaccine and did not complete the two-dose schedule. This is an indicator used for multi-dose vaccines and was calculated using the formula: Dropout rate = [(number of initial vaccine doses - number of ending vaccine doses) ÷ (number of initial vaccine doses) × 100]. The following parameters were used to analyze the immunization dropout rate: (i) low dropout rate < 5%; (ii) mean dropout rate ≥ 5 and < 10%; and (iii) high dropout rate ≥ 10% [29, 30].

## Results

A total of 666 adolescent girls and 623 mothers/guardians participated in the study. Forty-three mothers/guardians were responsible for more than one adolescent girl. Table 1 shows the sociodemographic and behavioral characteristics of the participants.

**Table 1:** Sociodemographic and behavioral characteristics of adolescent girls and their mothers/guardians

Characteristics	Adolescent girls		Mothers/Guardians	
	Categories	n (%)	Categories	n (%)
Age*	11 years	162 (24.3)	≤ 35 years	208 (33.5)
	12 years	199 (29.9)	36-49 years	308 (49.5)
	≥13 years	305 (45.8)	≥50 years	106 (17.0)
Educational level**	≤ 6th grade of elementary school	189 (28.4)	Illiterate/Elementary school (incomplete or complete)	336 (54.1)
	7th–8th grades of middle school	398 (59.9)	High school (incomplete or complete)	235 (37.8)
	9th grade of middle school - 1st year of high school	78 (11.7)	Higher education (incomplete or complete)	50 (8.1)
Residence area	Rural	270 (40.5)	Rural	244 (39.2)
	Urban	396 (59.5)	Urban	379 (60.8)
Household income (monthly)***	–	–	≤US$750	482 (80.2)
	–	–	US$751–1500	103 (17.1)
	–	–	>US$1500	16 (2.7)
Religion	No	48 (7.2)	No	24 (3.8)
	Catholic	500 (75.1)	Catholic	473 (75.9)
	Others	118 (17.7)	Others	126 (20.2)
Marital Status	–	–	Married	369 (59.2)
	–	–	Partner	83 (13.3)
	–	–	Single	94 (15.1)
	–	–	Widowed	33 (5.3)
	–	–	Separated/Divorced	44 (7.1)
Boyfriend	No	577 (86.6)	–	–
	Yes	89 (13.4)	–	–
Sexual intercourse****	No	654 (98.4)	No	3 (0.5)
	Yes	11 (1.6)	Yes	595 (99.5)
Age of 1st sexual intercourse*****	9-12 years	2 (18.2)	≤ 15 years	137 (23.0)
	13-14 years	7 (63.6)	16-20 years	303 (50.9)
	15 years	2 (18.2)	> 20 years	134 (22.5)
			Did not know	21 (3.6)
Number of sexual partners*****	1	8 (72.7)	1	282 (48.5)
	2	1 (9.1)	≥ 2	274 (47.2)
	≥ 3	2 (18.2)	Did not know	25 (4.3)
Vaccine uptake	None dose	22 (3.3)	–	–
	One dose	208 (31.2)	–	–
	Two doses	436 (65.5)	–	–

* Some adolescent girls were interviewed with more than 13 years old because the recruitment period was from 2014 to 2016. One guardian did not report the age

** One adolescent girl was not attending school. Two guardians did not know their level of education

*** Twenty-two guardians did not report on household income

**** One adolescent girl and twenty-five mothers/guardians did not report about sexual intercourse

***** Variables evaluated only for participants who reported having started sexual intercourse. Fourteen mothers/guardians did not report on the number of sexual partners

Mean age of adolescent girls was 12.4 ± 1.0 years. It was observed that 59.9% (*n* = 398/665) of adolescent girls were in the 7th and 8th grades of middle school and 59.5% (*n* = 396/666) resided in urban area. Most of them followed catholic religion (*n* = 500/666; 75.1). Only 1.6% (*n* = 11/666) of adolescent girls had sexual intercourse. Furthermore, 65.5% (*n* = 436/666) of them received two-dose of HPV vaccine (Table 1).

The age range of guardians was 18 to 77 years, with mean of 40.6 ± 10.1 years. Most were mothers (*n* = 489/623; 78.5%), had elementary school or less (*n* = 336/621; 54.1%), and followed catholic religion (*n* = 473/623; 75.9%). Regarding household income, 80.2% (*n* = 482/601) of mothers/guardians reported to earn US$750 or less per month (Table 1). Concerning sexual life, few mothers/guardians (*n* = 32/623; 5.1%) reported to have received a diagnosis of a STI, with the most common being HPV infection (*n* = 9/32; 28.1%). Among the 489 mothers in this study, 4.3% (*n* = 21/489) never had a Pap smear. Regarding the number of exams, 30.8% (*n* = 144/468) of mothers had up to five Pap smears, 72.6% (*n* = 340/468) said they performed it annually, and 69.2% (*n* = 324/468) had performed in the last 12 months or less (data not shown).

### Knowledge about HPV vaccine

Mothers/guardians answered correctly the questions for the knowledge evaluation more often than adolescent girls. When asked about the concept of the vaccine, 71.5% (*n* = 476/666) of adolescent girls and 79.9% (*n* = 498/623) of the mothers/guardians correctly cited its prophylactic nature. Regarding the HPV vaccine, almost half of adolescent girls (*n* = 330/666; 49.5%) knew the number of doses adopted by the official immunization schedule. However, only 11.6% (*n* = 77/666) knew about the correct interval between doses, and 39.2% (*n* = 244/623) of mothers/guardians correctly identified the HPV vaccination target population. In addition, only 18.3% (*n* = 122/666) of adolescent girls and 26% (*n* = 162/623) of mothers/guardians did not believe that HPV vaccine prevents other STIs; some participants cited that HPV vaccine might prevent the Human Immunodeficiency Virus Infection (HIV) and syphilis (Table 2). Concerning the main sources of information about the HPV vaccine, 27.5% (*n* = 183/666) of adolescent girls and 36.4% (*n* = 227/623) of mothers/guardians reported that information was obtained from television or radio. Adolescent girls also obtained information from schools (*n* = 149/666; 22.4%), while health care providers were poorly cited by both adolescent girls (*n* = 80/666; 12%) and mothers/guardians (*n* = 103/623; 16.5%) (data not shown).

**Table 2:** Frequency of correct answers knowledge assessment about HPV, HPV vaccine, and cervical cancer of adolescent girls and mother/guardians

Questions^a^	Adolescent girls n (%)	Mothers/Guardians n (%)
Knowledge about HPV vaccine		
1- What are vaccines?	476 (71.5)	498 (79.9)
2- How many doses of HPV vaccine should be given?	330 (49.5)	–
3- What is the interval between HPV vaccine doses?	77 (11.6)	–
4- Who should get HPV vaccine?	–	244 (39.2)
5- Does HPV vaccine prevent other sexually transmitted infections?	122 (18.3)	162 (26.0)
Knowledge about HPV and cervical cancer		
6- What is a sexually transmitted infection?	199 (29.9)	–
7- What is the main transmission route of HPV?	98 (14.7)	242 (38.8)
8- What should women do to prevent HPV infection?	158 (23.3)	–
9- Does HPV infection have visible signs and symptoms?	125 (18.8)	159 (25.5)
10- What are the signs and symptoms of HPV infection?	7 (1.0)	36 (5.8)
11- Is HPV infection common?	169 (25.4)	112 (18.0)
12- What kinds of problems does infection with HPV cause?	89 (13.4)	204 (32.7)
13- What causes cervical cancer?	14 (2.1)	56 (9.0)
14- Why should we prevent HPV infection?	55 (8.3)	–
15- What is a Pap smear?	–	498 (79.9)

^a^Answers rated as correct: 1- Prevention of disease/To stay protected/Immunity. 2- Two-dose or three-dose. 3–0-6 months. 4- Adolescent girls 11-13 years old or 9-13 years old. 5- No/Only against HPV/Each disease has a specific vaccine. 6- Disease transmitted through sexual intercourse/Disease that is caught through sex and may be transmitted to someone else. 7- Sexual intercourse. 8- Get the vaccine/Avoid multiple sexual partners/Use a condom. 9- No/Asymptomatic/Silent disease. 10- Genital warts. 1 1- Yes. 12- Cancer/Cervical cancer/Uterine cervix disease/Warts genital. 13- HPV infection. 14- Prevent cancer/Prevent HPV/Prevent cervical cancer. 15- Examination to prevent cervical cancer/Collection of material, secretion or liquid

### Knowledge about HPV and cervical cancer

Few adolescent girls (*n* = 199/666; 29.9%) were able to explain what STI is. Consequently, more mothers/guardians (*n* = 242/623; 38.8%) than adolescent girls (*n* = 98/666; 14.7%) correctly indicated that HPV is mainly transmitted through sexual contact. On the issue of what should be done to prevent HPV infection, 23.3% (*n* = 158/666) of adolescent girls cited vaccination, condom use, and/or avoid having multiple sexual partners (Table 2). Therefore, 11.6% (*n* = 77/666) of adolescent girls limited prevention to receiving the HPV vaccine, and 12% (*n* = 80/666) indicated condom use as the primary method of HPV prevention (data not shown).

The two groups were also unaware of signs and symptoms of HPV infection, as only 1.0% (n = 7/666) of adolescent girls and 5.8% (*n* = 36/623) of mothers/guardians mentioned that genital warts may be a visible sign of infection. Furthermore, few adolescent girls (*n* = 169/666; 25.4%) and mothers/guardians (*n* = 112/623; 18.0%) knew that HPV infection is common (Table 2).

Awareness of what HPV infection can cause was another important point. However, only 13.4% (*n* = 89/666) of adolescent girls and 32.7% (*n* = 204/623) of mothers/guardians showed knowledge about the issue. Consequently, few adolescent girls (*n* = 14/666; 2.1%) and mothers/guardians (*n* = 56/623; 9.0%) were able to recognize the relationship between HPV infection and occurrence of cervical cancer, and less than 9.0% (*n* = 55/666) of adolescent girls knew why it is important to prevent HPV infection. On the other hand, most mothers/guardians (*n* = 498/623; 79.9%) correctly described how the Pap smear is performed and knew its purpose (Table 2).

### Comparison between the knowledge of adolescent girls and mothers/guardians

**Fig. 1:**
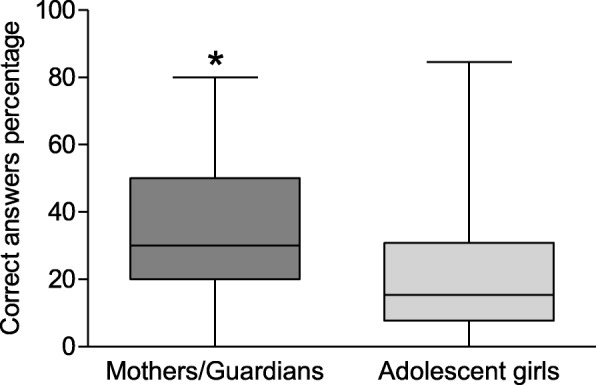
Distribution of the percentage of correct answers given by mothers/guardians and adolescent girls to knowledge assessment questions about HPV, HPV vaccine, and cervical cancer. The * represents the significant difference between the knowledge grade of mothers/guardians and adolescent girls

The knowledge score was determined based on the number of correct answers. Most of adolescent girls (*n* = 512/666; 76.9%) and mothers/guardians (*n* = 497/623; 79.8%) presented low knowledge on the subject. Comparing the percentage of correct answers between groups showed that mothers/guardians presented the highest index, with 30% (Q1 = 20%; Q3 = 50%) as the median of correct answers. This percentage was significantly higher (*p* < 0.0001) than results of adolescent girls, who scored a median of correct answers of 15.4% (Q1 = 7.7%; Q3 = 30.8%) (Fig. 1).

### Assessment of factors related to knowledge

The factors that could influence knowledge about HPV and cervical cancer for adolescent girls that showed to be significant in the univariate analysis (*p* < 0.25) included the variables: age, educational level, religion, and household income (reported by mother/guardian) (Table 3). The variable educational level was not included in multivariate analysis because it is collinear with the variable age. After successive removal of variables, the following factors were associated with risk of having low knowledge in the multivariate analysis: (i) age of 12 years [IRR 1.2 (95% CI 1. 1-1.3)] and age of 11 years [IRR 1.3 (95% CI (1. 2-1.4)], when compared to age of 13 years or more; (ii) household income lower or equal than US$750 per month [IRR 1.7 (95% CI 1. 1-2.6)] and household income between US$751 and US$1500 per month [IRR 1.6 (95% CI 1.0–2.6)] when compared to household income higher than US$1500 per month (Table 3).

**Table 3:** Univariate and multivariate analysis of factors associated with adolescent girls and their mothers/guardians’ knowledge about HPV and cervical cancer

Populations/Characteristics	Knowledge		IRR (95%CI)^a^	*p*-value	IRR (95%CI)^b^	*p*-value
	Low n (%)	High n (%)				
Adolescent girls						
Age						
≥ 13 years	206 (67.5)	99 (32.5)	1	–	1	–
12 years	161 (80.9)	38 (19.1)	1.2 (1. 1-1.3)	0.001	1.2 (1.1–1.3)	0.002
11 years	145 (89.5)	17 (10.5)	1.3 (1. 2-1.4)	0.000	1.3 (1. 2-1.4)	0.000
Educational level*						
9th grade of middle school - 1st year of high school	40 (51.3)	38 (48.7)	1	–	–	–
7th–8th grades of middle school	300 (75.4)	98 (24.6)	1.5 (1. 2-1.8)	0.001	–	–
≤ 6th grade of elementary school	171 (90.5)	18 (9.5)	1.8 (1. 4-2.2)	0.000	–	–
Religion						
Catholic	373 (74.6)	127 (25.4)	1	–	–	–
Others	97 (82.2)	21 (17.8)	1.1 (1.0–1.2)	0.053	–	–
No	42 (87.5)	6 (12.5)	1.2 (1.0–1.3)	0.008	–	–
Household income (monthly)**						
> US$1500	9 (47.4)	10 (52.6)	1	–	1	–
≤ US$750	401 (77.9)	114 (22.1)	1.6 (1.0–2.6)	0.041	1.7 (1. 1-2.6)	0.024
US$751–1500	83 (76.1)	26 (23.9)	1.6 (1.0–2.6)	0.055	1.6 (1.0–2.6)	0.032
Mothers/Guardians						
Educational level***						
Higher education (incomplete or complete)	27 (54.0)	23 (46.0)	1	–	1	–
High school (incomplete or complete)	166 (70.6)	69 (29.4)	1.3 (1.0–1.7)	0.050	1.2 (0. 9-1.6)	0.190
Illiterate/Elementary school (incomplete or complete)	302 (89.9)	34 (10.1)	1.7 (1. 3-2.2)	0.000	1.5 (1. 2-2.0)	0.003
Residence area						
Urban	290 (76.5)	89 (23.5)	1	–	–	–
Rural	207 (84.8)	37(15.2)	1.1 (1.0–1.2)	0.009	–	–
Household income (monthly)****						
US$751–1500	66 (64.1)	37 (35.9)	1	–	1	–
> US$1500	9 (56.3)	7 (43.7)	0.9 (0. 6-1.4)	0.576	1.0 (0. 6-1.6)	0.936
≤ US$750	402 (83.4)	80 (16.6)	1.3 (1. 1-1.5)	0.001	1.2 (1.0–1.4)	0.023
Marital Status						
Married	288 (78.1)	81 (21.9)	1	–	–	–
Partner	71 (85.5)	12 (14.5)	1.1 (1.0–1.2)	0.083	–	–
Single	76 (80.9)	18 (19.1)	1.0 (0. 9-1.2)	0.538	–	–
Widowed	27 (81.8)	6 (18.2)	1.0 (0. 9-1.2)	0.586	–	–
Separated/Divorced	35 (79.6)	9 (20.4)	1.0 (0. 9-1.2)	0.815	–	–
Religion						
Catholic	383 (81.0)	90 (19.0)	1	–	–	–
Others	99 (78.6)	27 (21.4)	1.0 (0. 9-1.1)	0.560	–	–
No	15 (65.5)	9 (37.5)	0.8 (0. 6-1.1)	0.105	–	–

^a^Univariate analysis, ^b^Multivariate analysis

* One adolescent girl was not attending school. The variable educational level was not included in the multivariate analysis because it is collinear the variable age

** The household income of adolescent girl was reported by mother/guardian

*** Two guardians did not know their level of education

**** Twenty-two guardians did not report on household income

For mothers/guardians, the variables educational level, residence area, household income, to be married and religion obtained *p* value < 0.25 by the univariate analysis. Thus, factors associated with risk of low knowledge of mothers/guardians were: (i) to be illiterate, not having or having completed elementary school [IRR 1.5 (95% CI 1. 2-2.0)] versus having or not having complete higher education; (ii) household income lower or equal than US$750 per month [IRR 1.2 (95% CI 1.0–1.4)], if compared with household income between US$751 and US$1500 per month (Table 3).

About one third (*n* = 208/666; 31.2%) of the adolescent girls received only one dose of the HPV vaccine, and 3.3% (*n* = 22/666) did not receive any dose at all of the vaccine (Table 1). It was also evaluated the association between vaccination uptake and knowledge of both adolescent girls [IRR 1.0 (95% CI 0. 9-1.1)] and mothers/guardians [IRR 1.0 (95% CI 0. 9-1.0)], and results indicated no significant association.

### Immunization dropout rate among adolescent girls

The dropout rate was determined by the proportion of adolescent girls who received the first dose of the vaccine and dropped out before completing the two-dose vaccination schedule. A high rate of uptake of the initial dose of HPV vaccine (96.7%) was observed. However, only 65.5% of adolescents completed the schedule, which is lower than 80% goal recommended by the Brazilian Ministry of Health [15]. Thus, the dropout rate in our sample was high, at 32.3%.

## Discussion

The present study shows that knowledge about HPV and cervical cancer is low for Ouro Preto’s adolescent girls and their mothers/guardians. However, based upon the percentage of correct answers, it was observed that, as expected, mothers/guardians had more knowledge than adolescent girls. Sociodemographic factors, such as age, educational level, and household income interfered in the grade of knowledge of the participants about the subject. HPV immunization dropout rate was high (32.3%), but there was no association with low knowledge.

Most of the participants were unaware of the causal relationship between HPV and cervical cancer or between HPV and the development of genital warts. In addition, adolescent girls and mothers/guardians did not know that HPV infection is common, and they had low knowledge about signs and symptoms, and the route of transmission of this infection. Similar results were found in a study that analyzed the knowledge of women in the city of Natal, capital of the northeastern state of Rio Grande do Norte, Brazil, in which few interviewees cited correctly the mode of transmission of HPV, the signs and symptoms of virus infection, and correlated HPV infection with the occurrence of cervical lesions [31]. In contrast, 74.1% of adolescent girls and 67.6% of their mothers in Hong Kong correlated HPV infection with cervical cancer [19].

In our sample, higher knowledge was observed concerning the broader concept of vaccine and, consequently, its prophylactic purpose. However, participants had a superficial knowledge about the specifics of the HPV vaccine, such as the recommended age group for vaccination, and number and interval between doses, which was also evidenced in other studies [17, 22]. Moreover, 11.6% of adolescent girls understood that prevention of HPV infection was restricted to vaccination. This appreciation of HPV vaccine may be a reflection of nationwide immunization campaign, and also of confidence of the Brazilian in the efficacy of immunobiologicals. It is known that frequent use of condoms is associated with decreased rates of HPV infection as well as other STIs [32]. However, few adolescent girls (12%) in this study considered condom use as primary method of HPV prevention. Therefore, vaccine may generate a false sense of complete protection by reducing care that leads to HPV prevention.

Another important topic in HPV prevention and cervical cancer control is the Pap smear. Among mothers in this study, approximately 95% reported having had the test at least once in their lifetime. Differently, Lima et al. [31] showed 87% coverage of the Pap smear in a study involving women in the city of Natal, Rio Grande do Norte, Brazil. In the same study, 69.4% of women performed Pap smear with a frequency of at least once every three years, as recommended in Brazil, which was lower than mothers rate of the present study (72.6%), who reported performing the test annually. In Brazil, in general, there is no organized screening program; thus, opportunistic testing predominates, in which while some women go without screening others are over-screened, as occurred with the mothers in our study. To improve the cost-benefit ratio, while increasing the coverage of the target population and decreasing cervical cancer rates, it is necessary proper implementation of early detection of cervical cancer strategies as indicated by the Brazilian Guidelines for Cervical Cancer Screening [33]. Regarding knowledge about cervical cancer screening, results showed that the most of mothers/guardians (79.9%) interviewed the current study in Ouro Preto had a higher level of knowledge than women of the city of Natal, as only 47% of Natal’s women had medium or high level of knowledge about Pap smear [31]. Although mothers/guardians recognize the importance of Pap smear to prevent cervical cancer, the fact that participants do not associate HPV infection with the development of cervical lesions may be a barrier to reduce cases of this neoplasm. Thus, there is a need to reinforce the relevance of Pap smear for early detection of cervical lesions.

Several socioeconomic, demographic, and behavioral factors may interfere with the knowledge about HPV and cervical cancer [23, 31, 34]. Having higher education, and higher household income had a positive influence on the knowledge. Indeed, we observed that older adolescents attending more advanced school levels and mothers holding a university degree and higher family income had better knowledge regarding HPV infection and prevention. These results corroborate Abreu et al., who showed that participants who reported high knowledge about HPV had a higher level of schooling and average family income (*p* < 0.001) [35]. Individuals with higher socioeconomic status possibly have more access to information, and consequently could be more aware regarding HPV infection and prevention measures [21, 23, 34, 35]. The main source of information cited by participants in our study was television/radio. However, such media is not considered the most appropriate or it is not enough to improve knowledge and lead to prevention behavior [35, 36]. Moreover, low level of schooling can make it difficult to understand the content transmitted by these or other sources of information [21, 35, 36]. In our sample, few participants reported that they obtained information about the HPV vaccine from health care providers. Previous studies have shown that health providers play a key role in improving the level of knowledge and acceptability of the HPV vaccine. Health care providers should promote communication about HPV infection and its prevention, and thus bring awareness on vaccine effectiveness [34, 36, 37].

Decreasing trends in HPV immunization uptake have been observed in Brazil. According to official data, from 2014 to early 2018, only 48.7% of adolescent girls aged 9 to 14 years completed a two-dose schedule [16]. This coverage rate is below the 80% goal recommended by the Brazilian Ministry of Health for HPV vaccination [15]. In this study, only 65.5% of adolescent girls adhered to HPV vaccination. Previous research described that adequate knowledge about HPV infection and awareness of prevention against this virus result in higher vaccine acceptance rates [18, 21, 23]. However, in our study, HPV knowledge was not associated with vaccine uptake. This may have occurred because a significant portion of participants presented important knowledge deficiencies on the subject. Thus, both adolescent girls who completed the vaccination schedule, and those who did not complete had low knowledge regarding HPV infection. In 2016, a study conducted in the northern state of Roraima, Brazil, showed a relatively high rate of non-adherence to HPV vaccination (17.3%). The authors reported that the lack of HPV knowledge negatively influenced the acceptance of HPV vaccine by parents/guardians of girls [18]. Other studies reported that mothers/guardians play a key role in the decision-making regarding vaccination uptake [23, 38–40]. Thus, there is a clear need for the optimization and qualification of the information that will be transmitted to the population regarding HPV and cervical cancer. Additional improvements in public health strategies for HPV vaccine and cervical cancer should include educational interventions tailored to different audiences with the support of health care professionals and teachers. These are considered to be potentially influential groups to promote the increase of HPV vaccine uptake [18, 19, 38, 41].

This study was conducted in a small town, so caution should be exercised in generalizing the findings to other locations. However, the number of adolescent girls and mothers/guardians interviewed was high and recruitment occurred in both urban and rural areas. In addition, it is a region with a large movement of people and the results of this study are corroborated by similar research conducted in Brazil and in other countries. Thus, the data obtained herein may guide public health actions aimed to prevent HPV infection in places with a similar population profile.

Considering that a wide interval was used to define “high knowledge”, both adolescent girls and their mothers/guardians have reached a very low knowledge score. Thus, this study points to the need of implementing educational initiatives on HPV and cervical cancer. For guarantee the effectiveness of educational interventions, it is essential to look at socio-cultural diversities, the ability of understanding, and access to information of each population group [21, 34]. Furthermore, the combination of different means of communication especially schools, health care providers, and social networks, has been shown to be effective in increasing the understanding HPV infection and vaccine and induce changes of individual behaviors [36, 42].

## Conclusion

Adolescent girls and mothers/guardians presented low knowledge about HPV and cervical cancer. However, despite the low knowledge, mothers/guardians demonstrated better understanding of the subject than the adolescent girls. High knowledge was associated with higher educational level and higher household income. In addition to low knowledge, we observed a high immunization dropout rate of HPV vaccination among adolescent girls. These findings reinforce the need for more effective educational actions regarding HPV and cervical cancer, especially in low-income population to promote awareness and prevention of this infection.

## Data Availability

The datasets used and/or analysed during the current study are available from the corresponding author on reasonable request.

## Abbreviations

CI: Confidence interval
HIV: Human Immunodeficiency Virus
HPV: Human Papillomavirus
IRR: Incidence rate ratio
Q: Quartile
STI: Sexually transmitted infection
